# Are B‐symptoms more reliable prognostic indicators than substage in canine nodal diffuse large B‐cell lymphoma

**DOI:** 10.1111/vco.12661

**Published:** 2020-11-23

**Authors:** Ondřej Škor, Ludmila Bicanová, Birgitt Wolfesberger, Andrea Fuchs‐Baumgartinger, Barbara Ruetgen, Marie Štěrbová, Ilse Schwendenwein, Miriam Kleiter

**Affiliations:** ^1^ Department of Companion Animals and Horses Vetmeduni Vienna Austria; ^2^ Department for Pathobiology Vetmeduni Vienna Austria; ^3^ Small Animal Clinic University of Veterinary and Pharmaceutical Sciences Brno Czech Republic

**Keywords:** B‐symptoms, canine nodal diffuse large B‐cell lymphoma, clinical signs, prognosis, substage, survival, treatment response

## Abstract

In humans B‐symptoms refer to systemic symptoms of lymphoma such as fever, weight loss, and night sweats and influence the prognosis of patients. In canine lymphoma, substage B is used to describe any clinical sign observed. Aim of the retrospective study was to compare the prognostic value of substage B with B‐symptoms to predict treatment response and survival in canine nodal diffuse large B‐cell lymphoma. Affected dogs treated with CHOP chemotherapy between 2008 and 2019 were included. B‐symptoms were defined by weight loss greater than 10% of normal weight, fever and the occurrence of unexplained resting tachypnoea substituted human night sweats. Substage B was defined as any symptoms but lymphadenopathy. Fifty‐five cases were included. B‐symptoms were present in 20/55 (36%) and substage B in 40/55 (74%) patients. No significant associations between B‐symptoms or substage B and weight, sex, breed, WHO stage and lymphoma grade were found. Treatment response was negatively associated with both substage B (*P = .02*) and B‐symptoms (*P = .001*). B‐symptoms significantly decreased progression free survival (PFS) (95 vs 330 days, *P = .001*) and lymphoma specific survival (LSS) (160 vs 462 days, *P = .001*). Data showed that B‐symptoms might be a more reliable prognostic indicator than substage B in canine nodal diffuse large B‐cell lymphoma. Prospective studies assessing B‐symptoms in a larger cohort of patients and in other common lymphoma types are warranted. The abstract was presented at the fourth meeting of the European Canine Lymphoma Network Group in Lugano, 22 June 2019 and published in the proceeding of the meeting on the page 26.

## 1 INTRODUCTION

Diffuse large B‐cell lymphoma (DLBCL) is the most common subtype of non‐Hodgkin lymphoma in humans and dogs. Approximately 50% of all newly diagnosed cases and more than 80% of aggressive lymphomas are DLBCL.^1^, ^2^ Recent insights into the pathogenesis of DLBCL suggest that it is a heterogeneous group rather than a single entity.^1^ Different morphologic variants are recognized, a variety of molecular and genetic abnormalities are present, and patients exhibit a wide range of clinical presentations and outcomes.^3^

The CHOP (cyclophosphamide, doxorubicin, vincristine, and prednisolone) chemotherapy protocol has been the mainstay of therapy for several decades.^4^ Attempts to improve outcomes with more aggressive chemotherapy failed to show additional benefit.^5^, ^6^ The recent development of chemo‐immunotherapy led to transformation of treatment practices in veterinary and human oncology and has improved outcome.^4^, ^7^ Although DLBCL is one of the most common lymphoma subtypes in dogs and it is in most cases highly responsive to chemotherapy, not every dog responds with complete remission to treatment.^4^ Early identification of poor‐risk patients may allow for alternate treatment strategies to be considered.

One of the most widely reported prognostic indicators is lymphoma substage, which has been correlated with complete treatment response (CTR), progression‐free survival (PFS) and lymphoma‐specific survival (LSS) in several studies.^8^, ^9^ The World Health Organization TNM Classification of Tumours of Domestic Animals details has stated simply substage A as the absence and substage B as the presence of clinical signs.^10^ A recent study used a survey to query veterinary oncologists on the clinical criteria to define substage.^11^ Gastrointestinal, constitutional, respiratory, neurologic, metabolic and nutritional variables were pivotal in assigning clinical substage. For most factors, a mild to moderate severity of clinical signs was sufficient for substage B designation.^11^ However, any unrelated clinical sign could be interpreted as substage B rendering this system less reliable.

In human oncology, presence of B‐symptoms is used to refer to systemic clinical signs of lymphoma as fever, weight loss, and night sweats and this classification influences prognosis.^12^

Aim of our retrospective study was to compare the ability of substage B vs the presence of B‐symptoms to predict treatment response and survival in canine nodal DLBCL (nDLBCL). We hypothesized that the presence of B‐symptoms is superior to substage B in predicting outcome.

## 2 MATERIALS AND METHODS

### 2.1 Inclusion criteria

Previously treatment‐naïve dogs diagnosed with nDLBCL at the Vetmeduni Vienna (Austria) and the Veterinary University Brno (Czech Republic) between 2008 and 2019 were included in this retrospective study. Fifty‐five dogs from 113 patients with diagnosed lymphoma met the inclusion criteria: (a) confirmed nDLBCL by histopathology and immunohistochemistry, (b) no prior oncologic treatment including corticoids, (c) a standardized first‐line chemotherapy CHOP (combination of cyclophosphamide, doxorubicin, vincristine, and prednisolone), and (d) a standardized clinical staging at the time of the initial diagnosis (at least based on peripheral blood analyses, thoracic radiographs, and abdominal ultrasound).

### 2.2 Medical records review

Medical record data were reviewed and patient characteristics (age, breed, sex, weight), history, clinical symptoms, results of lymphoma staging, classification and grading, CTR and survival (PFS and LSS) were recorded. LSS was calculated from the date of diagnosis to the date of death related to the lymphoma. PFS was calculated from the date of diagnosis to the date of disease progression or recurrence. Follow‐up information was obtained from medical records and by phone conversations with referring veterinarians and pet owners. Since 2016 a lymphoma‐related questionnaire was used to assess history and signalment of patients. Dogs with primary extranodal or disseminated DLBCL were excluded from the study. Extranodal DLBCL was defined as lymphoma arising primarily outside of lymph nodes and spleen. Disseminated DLBCL was characterized as lymphoma involving broad spectrum of nodal and extranodal sites without evidence of primary origin.^13^ Dogs that were alive at the end of study or lost to follow‐up were censored at the date of their last contact.

### 2.3 Lymphoma staging and classification

Medical records were explored for staging data including assessment of tumour extent (peripheral blood analyses, thoracic radiography, abdominal ultrasonography, fine‐needle aspiration of liver, spleen and bone marrow and final stage based on the current World Health Organization (WHO) classification.^10^ According to the published consensus of the Veterinary Comparative Oncology Group (VCOG) on response evaluation criteria for peripheral nodal lymphoma in dogs, a bone marrow aspirate was only requested if there was initially a clinical indication in the peripheral blood count/smear (cytopenia of unknown origin and/or lymphocytosis).^14^

Ultrasonographic changes in homogeneity as well as echogenicity of liver and spleen were considered positive for lymphoma despite published literature.^15^

Cytological samples of liver and spleen were considered as positive for lymphoma involvement if more than ≥5% of nucleated cells consisted of large lymphocytes.^15^, ^16^ Bone marrow cytology was recorded as positive if more than 3% of large lymphocytes or aggregates of lymphocytes were observed.^17^ Dogs without peripheral blood analyses, thoracic radiographs and/or abdominal ultrasonography were excluded from the study.

### 2.4 Definition of substage and B‐symptoms

Substage A was defined as the absence of clinical signs but peripheral lymphadenopathy and substage B as presenting with any clinical signs of illness including gastrointestinal, constitutional, respiratory, neurologic, metabolic and nutritional symptoms.^11^ B‐symptoms were defined by unintentional weight loss of >10% of normal body weight over a period of six months or less, fever >39°C for more than three days without any evident cause other than lymphoma and human drenching night sweats were substituted with a presence of unexplained tachypnea at rest, that was defined as a higher frequency (panting) of breathing observed by owners in rest without any evident cause other than lymphoma.^12^ B‐symptoms have been in use for human lymphoma for decades and their accurate origin cannot be recalled from the published literature. The first documented adoption was established together with the Ann‐Arbor system.^18^ As there is no published clinical experience with B‐symptoms in veterinary literature, we adopted criteria being long‐term in use for humans.

### 2.5 WHO classification

WHO classification was performed from prospectively collected (n = 14) and archived samples (n = 41) in all included patients. Histopathology samples were reviewed by one experienced veterinary pathologist (A.F.B.) who was blinded to follow‐up data, to avoid observer bias. A peripheral enlarged lymph node was surgically removed, formalin‐fixed and paraffin embedded, stained with haematoxylin and eosin, and examined by the veterinary pathologist. For immunohistochemistry, a polyclonal antibody against CD3 (Dako A 0452), a monoclonal antibody against CD79a (Abcam ab 3121, clone HM 47/A9), and in a part of the cases a polyclonal antibody against CD20 (Spring Bioscience E 2560) were used on paraffin‐embedded sections. DLBCL was classified according to published adapted veterinary WHO classification for lymphoid neoplasia.^19^ The defining features of DLBCL were the diffuse arrangement of sheets of neoplastic B cells and the uniformly large nuclei (>two red cells in diameter) and a scant cytoplasm of neoplastic cells. Nuclei were usually round or rarely cleaved or indented.^19^ Lymphoma with 0 to 5 mitoses per high power field (HPF, 40x objective) were graded as grade I (low grade), with 6 to 10 mitoses per HPF as grade II (intermediate grade) and with more than 10 mitoses/HPF as grade III (high grade).^19^

### 2.6 First‐line chemotherapy treatment

All dogs were treated with a first‐line CHOP chemotherapy, consisting of cyclophosphamide (week 1, 5, 9, 13, and 17), doxorubicin (week 3, 7, 11, 15, and 19), vincristine (week 2, 4, 5, 9, 13, and 17) and prednisolone (week 1‐5).

### 2.7 Assessment of treatment response

Treatment response was evaluated by using the following VCOG criteria for response evaluation in peripheral nodal lymphoma in dogs: complete remission (CR), 100% reduction in size of all measurable disease; partial remission (PR), *>*30% but *<*100% reduction in size of all measurable disease; stable disease (SD) *<*30% reduction in size of all measurable disease with no change in size or *<*20% increase in size of all measurable disease; and progressive disease (PD), *>*20% increase in size of all measurable disease or the appearance of any new lesion.^14^ Response was evaluated at each therapeutic session and was required to last for at least 28 days as previously reported.^14^ For statistical analysis, CTR was defined as 100% reduction in size of all measurable and non‐measurable disease present at the end of the first‐line treatment.

### 2.8 Statistical analyses

Statistical analysis was performed with the statistical software package SPSS v.20 for Windows (IBM SPSS, IBM Corporation, Chicago, Illinois) and the statistics add in software for Microsoft Excel Analyse‐it (Analyse‐it, version 2.03, Leeds, UK).

Statistical analysis included the *t*‐test for normally distributed data and the Mann‐Whitney *U*‐test for non‐normally distributed or categorical data. Pearson *r* correlation was used to compare the relationship between linear‐related variables, and Spearman rank correlation between nonparametric variables.

PFS was calculated as the interval between initiation of treatment and disease progression or relapse, whereas LSS was measured as the interval between the day of primary diagnosis and lymphoma‐related death. Dogs lost to follow‐up or dead for lymphoma‐unrelated causes, as well as those still in complete remission at the end of the study, were censored from survival analysis. CTR was defined as the achievement of complete remission during and at the end of induction chemotherapy.

Survival curves were analysed with the Kaplan ‐ Meier method. Survival times are presented as median (range). Differences between Kaplan ‐ Meier curves were assessed by the log‐rank test. For all statistical analyses, a *P‐*value *<*.05 was considered to be significant. Variables found to display statistical significance at the 5% level were included in a multiple analysis using multiple Cox proportional hazard analyses method.

## 3 RESULTS

Fifty‐two dogs were included into this study. The median body weight of the dogs was 28 kg (range: 3‐55 kg) and the median age at the time of the initial examination of 8.9 years (range: 3‐15 years) (Table 1). Thirty dogs (55%) were females (25 spayed, 78% of all females) and 25 dogs (45%) were males (nine neutered, 39% of all males) (Table 1). Eleven dogs were mixed breed dogs, the remaining 44 dogs represented 21 breeds, including Rottweiler (n = 5), Border Collie (n = 5), Golden Retriever (n = 4), Berner Mountain's Dog (n = 4), and 17 other breeds each represented by one or two dogs. Substage and B‐symptoms were not associated with sex, breed, age, weight.

**TABLE 1:** Clinical characteristics of included dogs with nDLBCL

Clinical characteristic	Number (%)
Mean weight (range)	29 kg (3‐65 kg)
Mean age (range)	8.9 years (3‐15 years)
Sex	
Female	5 (9%)
Female spayed	25 (46%)
Male	16 (29%)
Male neutered	9 (16%)
WHO stage	
Stage II	1 (2%)
Stage III	10 (18%)
Stage IV	27 (49%)
Stage V	17 (31%)
WHO grade	
Grade I	12 (22%)
Grade II	23 (42%)
Grade III	20 (36%)
B‐symptoms:	
Absence	35 (64%)
Presence	20 (36%)
Substage:	
A	15 (29%)
B	40 (71%)
TR during first‐line chemotherapy	
Complete remission	35 (64%)
Euthanasia because of PD	3 (5%)
Rescue treatment because of PD	17 (31%)
DMAC	7 (13%)
LOPP	4 (7%)
Lomustine	3 (5%)
Rabacfosadine	2 (4%)
Chlorambucil	1 (2%)

*Note*: Substage A, absence of any clinical sign but lymphadenopathy; substage B, presence of any clinical sign; Values are presented as median (range) or number (%).

Abbreviations: DMAC, chemotherapy with combination of dexamethasone, melphalan, dactinomycin and cytarabine; LOPP, chemotherapy with combination of lomustine, vincristine, procarbasine and prednisolone; PD, progressive disease; TR, treatment response.

All dogs but one was presented with generalized peripheral lymphadenopathy and this was the reason for initial presentation (Table 2). One dog was presented with peripheral lymphadenopathy on cranial part of diaphragm (stage II). Because of the retrospective study design, no reliable information on disease duration could be retrieved. At the time of diagnosis, 15 dogs (29%) were asymptomatic (substage A), whereas the remaining 40 dogs (71%) showed systemic signs of illness (substage B) (Tables 1 and 2). Major observed symptoms were lethargy (n = 35), behavioural changes (n = 18), weakness (n = 14), hyporexia (n = 13), weight loss (n = 13), diarrhoea (n = 9), unexplained tachypnea at rest (n = 9), fever (n = 8), coughing (n = 8), sneezing (n = 7), ocular changes (n = 6), choke (n = 6), dyspnoe (n = 5), vomiting (n = 5), nasal discharge (n = 5), polyuria/polydipsia (n = 4) and bruising (n = 3) (Table 2). B‐symptoms were observed in 20 dogs (36%) and no B‐symptoms were detected in 35 dogs (64%) (Tables 1 and 2).

**TABLE 2:** Observed clinical symptoms in included dogs with nDLBCL

Clinical sign	Substage B (n = 40, 71%)	B‐symptoms (n = 20, 36%)
Lymphadenopathy (n = 55, 100%)	−	−
Lethargy (n = 35, 64%)	+	−
Behavioural changes (n = 18, 33%)	+	−
Weakness (n = 14, 25%)	+	−
Hyporexia (n = 13, 24%)	+	−
**Weight loss (n = 13, 24%)**	**+**	**+**
Diarrhoea (n = 9, 16%)	+	−
**Tachypnea in rest (n = 9, 16%)**	**+**	**+**
**Fever (n = 8, 15%)**	**+**	**+**
Coughing (n = 8, 15%)	+	−
Sneezing (n = 7, 13%)	+	−
Ocular changes (n = 6, 11%)	+	−
Choke (n = 6, 11%)	+	−
Dyspnoe (n = 5, 9%)	+	−
Vomiting (n = 5, 9%)	+	−
Nasal discharge (n = 5, 9%)	+	−
Polyuria/polydipsia (n = 4, 7%)	+	−
Bruising (n = 3, 5%)	+	−

*Note*: −, absence of substage B and/or B‐symptoms; +, presence substage B and/or B‐symptoms; Substage B, presence of any clinical sign but lymphadenopathy; clinical signs in bold show a presence of B‐symptoms; n, number of patients.

Thoracic radiography and abdominal ultrasound were performed in all 55 dogs including liver and/or spleen aspirates in 12 cases (22%). Bone marrow aspiration was performed in nine cases (16%) and revealed positive results in all cases. Splenic infiltration was recorded in 27 dogs (49%). The liver was considered infiltrated in 11 dogs (20%), as documented by sonographic changes and/or confirmative cytology. Peripheral blood was involved in 10 dogs. In addition to these, 14 (25%) dogs had also extranodal involvement other than peripheral blood, bone marrow or liver, lung infiltrates were most frequently recorded (n = 9) followed by intraocular (n = 3), dermal (n = 2), central nervous system (n = 1) and urinary bladder (n = 1) lesions. Based on the WHO staging system one dog (2%) had stage II, 10 (18%) stage III, 27 (49%) stage IV and 17 (31%) stage V disease (Table 1).

Histology and immunohistochemistry were performed in all cases. Involved lymph nodes were characterized by a diffuse growth pattern and loss of follicle‐related architecture. The capsule was documented to be thinned and taut. The greatest proportion of cells was large sized (two‐three times the red blood cell) defined as centroblasts and/or immunoblasts, with scant eosinophilic cytoplasm, round nucleus with multiple nucleoli (centroblasts) or a single prominent central nucleolus (immunoblasts). The mitotic activity of these cells was variable. There were 12 grade I, 23 grade II and 20 grade III cases (Table 1). Tingible body macrophages were present. Uniform immunohistochemical positivity of the neoplastic cells for CD79a and negativity for CD3 confirmed B‐cell origin.

All included dogs were treated with first‐line chemotherapy (CHOP). Thirty‐five (64%) dogs completed the planned treatment schedule in CR and three (5%) died/were euthanized due to PD. In the remaining dogs (n = 17, 31%) based on a progression during first‐line treatment, a rescue chemotherapy was started: seven were treated with a combination of dexamethasone, melphalan, D‐actinomycin and cytarabine (DMAC), four with a combination of lomustine, vincristine, procarbasine and prednisolone (LOPP), three with lomustine monotherapy, two with rabacfosadine and one with metronomic chlorambucil (Table 1).

From the group that reached CR after induction chemotherapy, 26 dogs (74%) were treated with rescue chemotherapy because of recurrence: 14 dogs received again a CHOP‐based protocol, whereas six patients were treated with DMAC, three with LOPP, two with lomustine monotherapy and one with rabacfosadine.

Median PFS was 188 days (range: 15‐1544 days) and median LSS was 259 days (range: 29‐1645 days). At the end of the study data analysis closure, nine dogs were alive, three lost from follow‐up, 36 dead because of lymphoma and seven from causes other than DLBCL. Other causes of death were renal (n = 2), cardiac (n = 2) failure and secondary neoplasia (n = 3). The median follow‐up period was 257 days (range: 25‐1634 days).

**TABLE 3:** Summary of Kaplan–Meier estimates in univariate PFS analysis for substage and B‐symptoms

	Event (n)	Cens. (n)	Mean	95% CI	Median	Range	*P* value
Substage A	10	5	436	26‐609	240	74‐910	.225
Substage B	30	10	394	217‐570	160	0‐1544	
Absence of BS	21	14	626	417‐836	332	15‐1544	**<.001**
Presence of BS	19	1	87	66‐107	91	0‐161	

*Note: P* values in bold show a statistically significant association.

Abbreviations: 95% CI, 95% confidence interval; BS, B‐symptoms; Cens., censored; n, number of cases; PFS, progression free survival.

**FIGURE 1:**
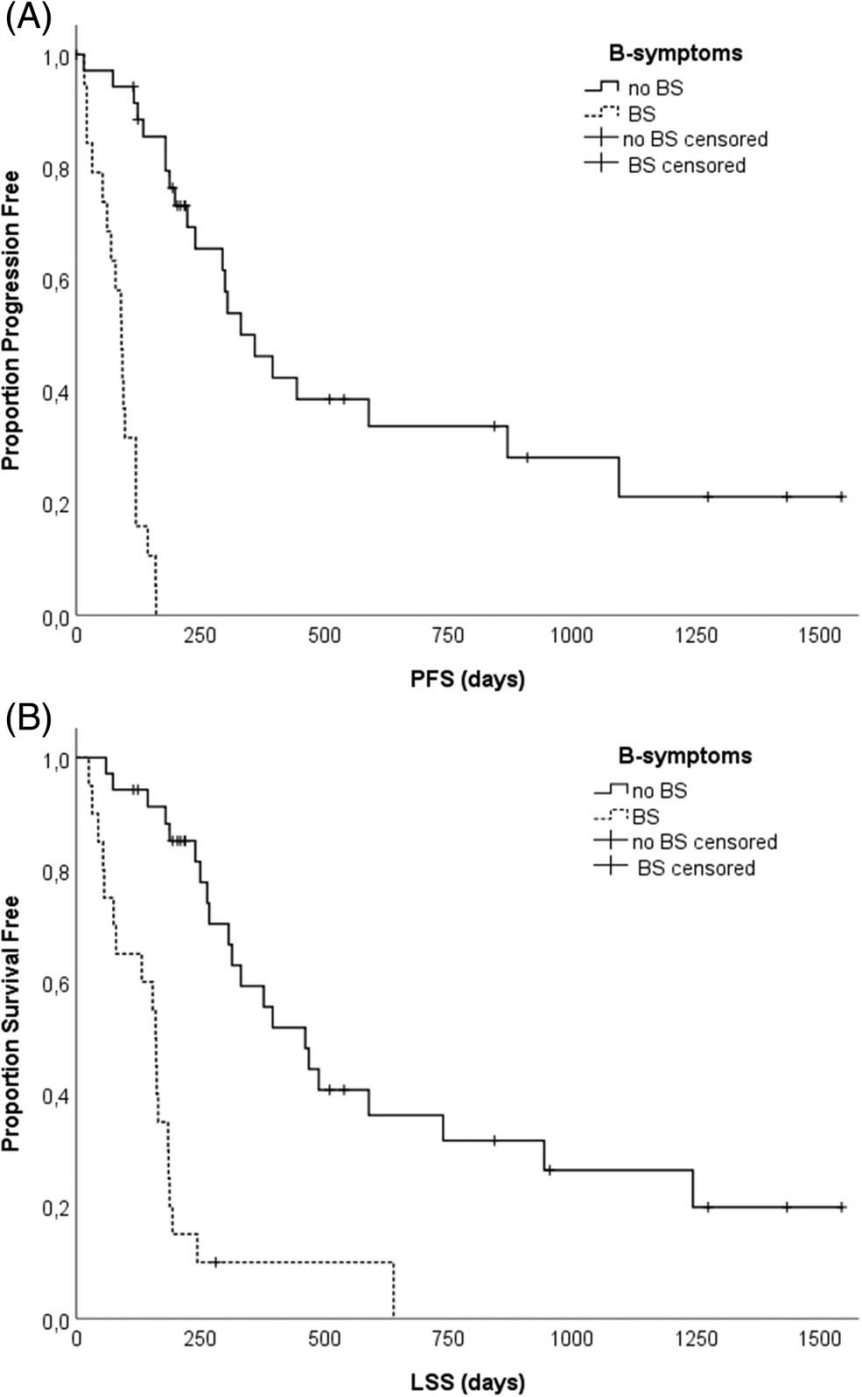
A, Kaplan–Meier progression‐free survival of the 55 canine patients with nDLBCL stratified according to the presence or absence of B‐symptoms: Presence of B‐symptoms was an independent predictor of inferior time to progression/tumour recurrence (*P = .001*). B, Kaplan–Meier lymphoma specific survival of the 55 canine patients with nDLBCL stratified according to the presence or absence of B‐symptoms: Presence of B‐symptoms was an independent predictor of inferior survival (*P = .001*)

The presence of B‐symptoms was significantly associated with shorter PFS and LSS (*P < .001* for both) (Table 3, Figure 1A,B). Median PFS for dogs with and without B‐symptoms was 91 (SD ± 10.2 days) vs 332 days (SD ± 58.7 days) respectively (Table 3). Median LSS for dogs with and without B‐symptoms was 162 (SD ± 7.8 days) vs 462 days (SD ± 77.5 days), respectively.

**FIGURE 2:**
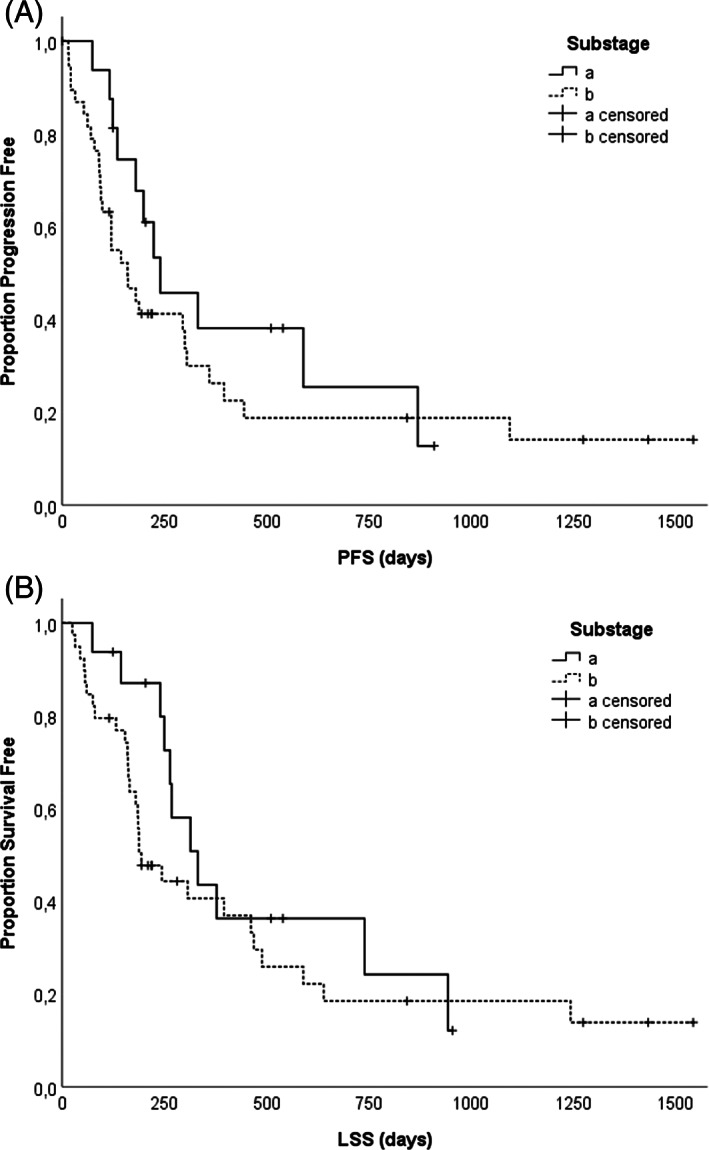
A, Kaplan–Meier progression‐free survival of the 55 canine patients with nDLBCL stratified according to the substage: Substage B was not linked with inferior time to progression/tumour recurrence (*P = .18*). B, Kaplan–Meier lymphoma specific survival of the 55 canine patients with nDLBCL stratified according to substage: Substage B was not associated with inferior survival (*P = .225*)

The presence of substage B was not associated with shorter PFS (*P = .18*) and LSS (*P = .23*) (Table 3, Figure 2A,B). Median PFS for symptomatic (substage B) and asymptomatic (substage A) dogs was 160 (SD ± 27.7 dogs) vs 240 days (SD ± 91.5 days) (Table 3). Median LSS for symptomatic and asymptomatic dogs was 188 (SD ± 34.6 days) vs 332 days (SD ± 65.3 days), respectively.

**TABLE 4:** Association of substage and B‐symptoms with complete treatment response

	CTR	Without CTR	% in CTR	OR	*P* value
Substage A	13	2	87%		***.02***
Substage B	21	19	53%	1.7	
Absence of BS	31	4	89%		***.001***
Presence of BS	3	17	15%	5.9	

*Note: P* values in bold show a statistically significant association.

Abbreviations: CTR, complete treatment response; OR, odds‐ratio.

The presence of B‐symptoms (*P < .001*), but not substage B (*P = .18*), WHO stage (*P = .7*) and WHO grade (*P = .89*) remained statistically significant variables for PFS in univariate analysis. The univariate Cox regression analysis confirmed that the presence of B‐symptoms (*P < .001*), but not substage B (*P = .23*), WHO stage (*P = .28*), WHO grade (*P = .9*) remained significant prognostic factors for LSS. The presence of B‐symptoms (*P < .001*) and substage B (*P = .02*), but not WHO stage (*P* = *.7*) and WHO grade (*P = .89*) remained statistically significant variables for CTR in univariate analysis. The presence of B‐symptoms remained an independent prognostic indicator for PFS (*P = .001*), LSS (*P = .001*), and CTR (*P = .001*) in multiple regression analysis (Table 5).

**TABLE 5:** Cox proportional hazards regression model showing the effect of independent variables on PFS in multiple analysis

Independent variable	Coefficient (β)	SE	*P* value
WHO grade	0.7	0.4	.13
WHO stage	−0.6	0.5	.14
Substage	0.7	0.5	.13
B symptoms	−3.5	0.6	**.001**

*Note: P* values in bold show a statistically significant association.

Abbreviations: PFS, progression free survival; WHO, World Health Organization.

## 4 DISCUSSION

This study compares the prognostic significance of clinical signs defined by substage and B‐symptoms on outcome of 55 dogs with histologically confirmed nDLBCL. Detailed information can be provided despite the retrospective nature of the study, because complete datasets regarding initial staging, treatment were available for all included dogs. Follow up data were available for all but three dogs. Canine nDLBCL is regarded as an aggressive lymphoid neoplasia mostly characterized by a painless generalized peripheral lymphadenopathy.^2^ nDLBCL is not a homogenous disease entity and its prognosis is highly variable.^1^

Clinical substage has been recognized as an attractive prognostic indicator due to its clinical simplicity.^8^ It is ascertainable with a patient history and physical examination and does not rely on advanced staging. It is expected that clinical signs reflect the aggressiveness of theunderlying disease, which determines survival.^11^

The published studies describing canine nDLBCL suggested that substage B may be associated with inferior survival.^8^, ^16^ The presence of B‐symptoms in dogs with lymphoma has been historically concluded to be low and authors are not aware of any veterinary study proving its prognostic relevance in canine nDLBCL or any other lymphoma subtype according to WHO classification.^20^

Our data indicate that the presence of B‐symptoms is more likely to be of prognostic significance than substage B. Data suggests that B‐symptoms represent more accurately the biologic aggressiveness of canine nDLBCL than substage B. Any mild to moderate clinical symptom may be considered as substage B.^11^ However, any symptom could be a consequence of any other concomitant disease which may lead to a classification error regarding substage B. The false positive assignment of these patients into substage B may decrease its statistical power as a predictor of prognosis in canine nDLBCL.

Substage B and B‐symptoms were not associated with age, sex and breed in our study group. In the multiple regression analyses, B‐symptoms remained an independent prognostic factor for survival and treatment response.

Limitations of the present study are the retrospective design and the relatively small number of cases. Dogs were selected based on the inclusion criteria and were therefore not necessarily representative of all dogs with nDLBCL. Some dogs might have suffered from other unidentified chronic diseases, causing alterations in their clinical symptoms.

One important limitation of our study was the absence of a detailed staging including liver, spleen and bone marrow aspiration in all dogs. This is in a contrast with published literature showing that an altered echogenicity may be due to causes different from lymphoma, and that normal‐appearing organs may be still infiltrated.^15^, ^21^ Another limitation is the lack of a standardized rescue treatment protocol as a result of variability in clinician's and owner's preferences, which could have introduced differences in the investigated parameters.

All data regarding B‐symptoms and substage B were retrieved from medical records and/or recent client communication. Tachypnoea was defined as increased frequency of breathing in rest without any cause other than lymphoma. Based on the retrospective design of the study, interpretation bias cannot be ruled out completely. However, all dogs presenting with tachypnea displayed also one or two other B‐symptoms (weight loss and/or fever) making its influence on presented results unlikely. Nevertheless, the respective relevance of these three signs within B‐symptoms alone and their combinations shall be further investigated in prospective studies with canine nDLBCL. We will suggest to set a timeframe for tachypnoea at rest for at least 3 days.

Strengths of the study are the long‐term follow‐up of a well‐defined group of dogs with nDLBCL treated with a standardized first‐line chemotherapy and low number of patients lost from the follow‐up.

In conclusion, this study demonstrated that B‐symptoms may be a better prognostic indicator than substage B in canine nDLBCL.

B‐symptoms remained an independent predictor of survival in the multiple regression analysis. Human studies proved that B‐symptoms are associated with increased concentrations of acute phase proteins and some proinflammatory cytokines.^12^, ^22^ Future prospective studies assessing B‐symptoms in larger cohort of DLBCL patients and their association with inflammatory proteins and cytokines are warranted.

## CONFLICT OF INTEREST

The authors declare no potential conflict of interest.

## Data Availability

Data sharing is not applicable to this article as no new data were created or analyzed in this study.
